# Overexpression of *TcNTPDase-1* Gene Increases Infectivity in Mice Infected with *Trypanosoma cruzi*

**DOI:** 10.3390/ijms232314661

**Published:** 2022-11-24

**Authors:** Natália Lins da Silva-Gomes, Leonardo Alexandre de Souza Ruivo, Claudia Moreira, Marcelo Meuser-Batista, Cristiane França da Silva, Denise da Gama Jaen Batista, Stênio Fragoso, Gabriel Melo de Oliveira, Maria de Nazaré Correia Soeiro, Otacilio C. Moreira

**Affiliations:** 1 Plataforma de PCR em Tempo Real RPT09A, Laboratório de Virologia Molecular-IOC/FIOCRUZ, Rio de Janeiro 21040-360, Brazil; 2 Laboratório de Biologia Molecular de Tripanossomatídeos-ICC/FIOCRUZ, Curitiba 81350-010, Brazil; 3 Laboratório de Educação Profissional em Técnicas Laboratoriais em Saúde, EPSJV/FIOCRUZ, Rio de Janeiro 21040-360, Brazil; 4 Laboratório de Biologia Celular-IOC/FIOCRUZ, Rio de Janeiro 21040-360, Brazil

**Keywords:** *Trypanosoma cruzi*, chagas disease, ecto-NTPDases, TcNTPDase, virulence

## Abstract

Ecto-nucleoside triphosphate diphosphohydrolases (NTPDases) are enzymes located on the surface of the *T. cruzi* plasma membrane, which hydrolyze a wide range of tri-/-diphosphate nucleosides. In this work, we used previously developed genetically modified strains of *Trypanosoma cruzi* (*T. cruzi*), hemi-knockout (KO +/−) and overexpressing (OE) the *TcNTPDase-1* gene to evaluate the parasite infectivity profile in a mouse model of acute infection (*n* = 6 mice per group). Our results showed significantly higher parasitemia and mortality, and lower weight in animals infected with parasites OE *TcNTPDase-1*, as compared to the infection with the wild type (WT) parasites. On the other hand, animals infected with (KO +/−) parasites showed no mortality during the 30-day trial and mouse weight was more similar to the non-infected (NI) animals. In addition, they had low parasitemia (45.7 times lower) when compared with parasites overexpressing *TcNTPDase-1* from the hemi-knockout (OE KO +/−) group. The hearts of animals infected with the OE KO +/− and OE parasites showed significantly larger regions of cardiac inflammation than those infected with the WT parasites (*p* < 0.001). Only animals infected with KO +/− did not show individual electrocardiographic changes during the period of experimentation. Together, our results expand the knowledge on the role of NTPDases in *T. cruzi* infectivity, reenforcing the potential of this enzyme as a chemotherapy target to treat Chagas disease (CD).

## 1. Introduction

American trypanosomiasis or Chagas disease (CD) is a neglected tropical disease caused by the flagellated protozoan *Trypanosoma cruzi* (*T. cruzi*) [1]. This parasite represents a serious public health problem, especially in Latin America, where the disease is endemic in 21 countries [1,2]. Moreover, it is relevant in other non-endemic regions, such as Europe, North America, and Asia due to the growing migratory movement of infected people in these areas, which allows for the distribution of this disease to other continents. It is estimated that about 6 to 7 million individuals from different countries in Latin America are infected with this disease. Additionally, due to the high number of people who remain undiagnosed or untreated, combined with the areas with remaining active transmission, an estimated 70 million people are at risk of infection, with about 14 thousand deaths associated with this disease every year [1,2]. Despite efforts from different governmental and non-governmental initiatives that resulted in the sharp decline in new acute cases, CD still presents many challenges, including the lack of prophylactic therapies and effective therapeutic regimens, especially for chronic patients [2,3]. The situation is further aggravated by the existence of drug-resistant strains. It is unknown whether this drug resistance is acquired or whether it would be a natural feature of the parasite [4]. The chemotherapy currently used is based on antiparasitic drugs, such as nifurtimox and benznidazole, which cause many side effects and present poor effectiveness on the later chronic phase of the disease [5,6]. Therefore, in this scenario, the search for new drugs and targets to chemotherapy is pivotal and many efforts have been made to develop new, more specific drugs for the treatment of CD. Molecules localized at the parasite plasma membrane appear to be good targets, since they could be more exposed to the drugs. As a result, studies related to the deeper knowledge of biology and biochemistry of trypanosomatids allow for the identification of different biochemical pathways and targets essential for the survival of these parasites, which can be considered promising targets for the treatment of this disease, such as ergosterol metabolism, cysteines proteinases, trypanothione reductases, pyrophosphatases, and the purine salvage pathway, where the role of ecto-nucleotidases as a virulence factor is postulated, among others [2,7].

Ecto-nucleotidases are enzymes that correspond to a wide and diverse class of catalytic proteins, which can be membrane-bound or secreted (soluble protein), with the active site facing the extracellular environment. Ecto-nucleotidase families include ecto-nucleoside triphosphate diphosphohydrolase (E-NTPDase/CD39/NTPDase-1), ecto-nucleotide pyrophosphatase/phosphodiesterase (E-NPP), and ecto-5′-nucleotidase (E-5′-nucleotidase/CD73) [8,9,10,11,12]. The class of E-NTPDases, also known as apyrase or CD39 family, are capable of hydrolyzing a variety of di- and triphosphate purine and pyrimidine nucleosides in different environments, including the extracellular one. All members of this family have five conserved regions, called conserved regions of apyrases (ACRs), which are extremely important for the formation of the active site of these enzymes [11]. Some amino acid residues are extremely conserved in ACRs, while others are more variable, and this variation is a possible determinant of the difference in specificity for the substrate that exists between the various enzymes that make up the family [8,13,14,15,16,17]. Another important difference between the isoforms of these enzymes is that some of them can hydrolyze the triphosphate nucleosides directly into monophosphates in a single step, releasing two inorganic phosphates (NTPDase-1). Other isoforms hydrolyze the triphosphate nucleosides releasing an inorganic phosphate and the diphosphate nucleoside, which are only hydrolyzed to monophosphates when they reach a certain concentration in the reaction medium. This characteristic allows for a transient increase in the concentration of nucleoside diphosphates to occur, which can be important for cell signaling [8,18].

Several studies have already demonstrated the importance of extracellular nucleotides and their participation in inflammation, platelet aggregation, and immune response, among other cellular processes [19,20]. ATP and other nucleotides, when accumulated in the extracellular environment, due to stress or cell damage, indicate a “danger signal” to the immune system, triggering an inflammatory response from the host [15]. Under these conditions, ATP can bind to P2 receptors, inducing the release of pro-inflammatory cytokines, such as IL-1β, IL-12, as well as the production of TNF-α [21,22]. On the other hand, adenosine (ADO), a nucleoside generated by ATP enzymatic catabolism, when binding to P1 type receptors, is recognized as an immunomodulatory molecule, and inhibits the release of pro-inflammatory cytokines, inducing the release of IL-10 [23]. Therefore, the combined action of the enzyme E-NTPDase (CD39), hydrolyzing ATP → ADP → AMP, and the enzyme 5′-nucleotidase (CD73), hydrolyzing AMP → ADO, is of great importance for the control of the level of extracellular ATP [21]. E-NTPDases have already been described in eukaryotic organisms [23] and in several parasitic protozoa, including trypanosomatids, with one of its main functions as the hydrolysis of tri- and diphosphate to monophosphates [24]. In this context, these enzymes may play a great role during ATP and ADO balance (pro-inflammatory and anti-inflammatory, respectively), allowing the parasite to escape from the vertebrate host’s immune system, through their ecto-NTPDase activity, deactivating the host defenses that involve extracellular ATP and ADP, such as platelet activation and cytolytic reactivation of T lymphocytes [25].

Moreover, a better understanding of the role of E-NTPDases in the process of infectivity and virulence of parasites still needs further investigation. Previously, studies carried out by Fietto, in 2004, showed the location of TcNTPDase-1 on the external surface of all evolutionary forms of *T. cruzi* [25]. Although it has not yet been well elucidated and needs further studies, decreased infectivity and virulence in protozoa due to successive passages in culture have been described in the literature for years [26,27,28,29]. In another report in 2010, De Souza et al. evaluated ecto-NTPDase activities in virulent (<15 passages) and avirulent (>100 passages) strains of L. *amazonensis*, aiming to correlate the role of this enzyme with the parasite virulence [27]. They observed that parasites with more than 100 passages in culture showed a reduction in the hydrolysis activity of ATP and ADP, while parasites with less than 15 passages presented the opposite result. In the same study, it was observed that the parasites (>100 passages) were less virulent in vivo, producing less damage and a lower host immune response [27].

Studies that tried to observe the enzymatic activity of ecto-ATPDase on the surface of different forms of trypanosomatids showed that axenic amastigotes and metacyclic promastigotes exhibited the highest ecto-ATPase activity and the epimastigote forms exhibited the lowest levels of enzymatic activity [15,30,31]. Furthermore, Paes-Vieira et al., 2018 compared the ecto-ATPase activity in the three life forms of *Leishmania* (L.) *amazonensis* using intact promastigote, amastigote, and metacyclic promastigote parasites. Using CD39 antibody Western blot analysis showed that NTPDase-1 was poorly expressed in the blood promastigote form and significantly increased in axenic amastigotes and reached the highest expression level in metacyclic promastigotes [26]. Trypanosomatids are not able to produce their own purines to carry out their metabolic activities [32,33]. Some studies suggest that the uptake of these exogenous purines is performed by plasma enzymes, such as NTPDases [32]. These data may indicate that the presence of NTPDases on the surface of different forms of trypanosomatids may have different functions. Therefore, it is possible that these enzymes have two main functions among the forms: (1) To regulate the host’s immune system and (2) to obtain exogenous purines. In the first case, the enzymes act by deactivating the defense responses, decreasing the levels of ATP and ADP, by extracellular forms (trypomastigote), since the immune system of the hosts is regulated by the levels of ATP and ADP, as well as by the ADP-dependent platelet aggregation [34,35]. In the second case, as trypanosomatids do not synthesize purines, the amastigote, which are replicating forms, use these enzymes to obtain the machinery necessary for their own metabolism [33].

In previous studies [36], using a reversible inhibitor of ecto-ATPase activity, we showed that the ecto-enzymes of *Leishmania* (L.) *amazonensis* act as facilitators of macrophage infection. In addition, other studies have observed that the expression of TcNTPDase-1 is modulated by the parasite, between different strains and clones, evolutionary forms, and under induced stress, reiterating the importance of this gene in *T. cruzi* [37]. In a previous study, to further understand the role of this enzyme in the adhesion and internalization steps of these parasites into the vertebrate host cells, we developed genetically modified strains of *T. cruzi*, hemi-knockout and overexpressing the *TcNTPDase-1* gene [38]. We observed that the in vitro infection in VERO cells with KO +/− and OE TcNTPDase-1 parasites, showed a significant reduction and increase, respectively, in the adhesion and endocytic indices, as well as in the total parasitic load of infected VERO cell lines [38].

Many efforts are still needed to better understand the role of ecto-NTPDases in parasites. The literature has shown that these enzymes are related to the trypanosomatid infectivity and virulence processes [37,39]. However, at present, most studies focused on the enzymatic activity of ecto-NTPDases in intact parasites or plasma membrane fractions [33,40]. Therefore, there is a lack of information on the specific contribution of each of these enzymes during these cellular processes and the use of mice models in CD has proved to be important to gain insight into aspects of disease progression in humans [41,42,43,44]. Therefore, presently, recombinant *T. cruzi* (Dm28c clone, Tc I) knocking-down or overexpressing the *TcNTPDase-1* gene (AY540630.1) was investigated, to evaluate the specific contribution of this enzyme in the parasite infectivity and virulence, using an in vivo mouse model of *T. cruzi* acute infection with metacyclic recombinant trypomastigotes.

## 2. Results

### 2.1. Experimental Design and Parasitological Evaluation

**Figure 1:**
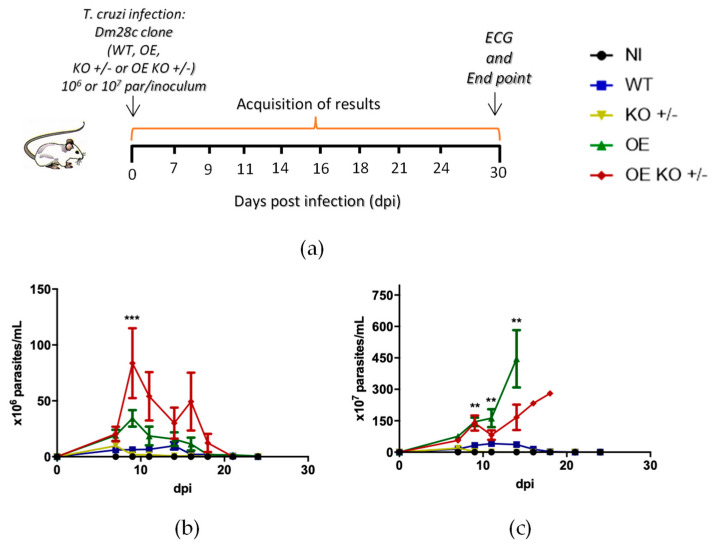
Experimental design and parasitological evaluation of extracellular forms of blood trypomastigotes from animals infected with *T. cruzi* hemi-knockout or overexpressing the *TcNTPDase-1* gene. Mice non-infected (NI) or infected with (wild type) WT, (overexpressed) OE, (hemi-knockout) KO +/−, and (overexpressed hemi-knockout) OE KO +/− were followed up to 24 days after infection. (**a**) Experimental design of infection of Swiss male mice inoculated intraperitoneally with 10^6^ or 10^7^ parasites/animal of Dm28c *T. cruzi* clones (WT, OE, KO +/− or OE KO +/−). (**b**) Parasitemia levels until 24 dpi, inoculum of 10^6^ par/animal. *** *p* < 0.001 at 9 dpi (OE KO +/− vs. KO +/−). (**c**) Parasitemia levels until 24 dpi, inoculum of 10^7^ par/animal. ** *p* < 0.01 at 9, 11, and 14 dpi (OE vs. KO +/−).

To validate our experimental mouse model of *T. cruzi* acute infection, male mice were infected using two parasite inoculums (10^6^ and 10^7^ parasites/animal, intraperitoneal route i.p.) and then followed for 30 days (Figure 1a). Parasitemia was assessed every 2 or 3 days from 7 days post-infection (dpi), weight data measured on days 11, 16, and 21 dpi. Only animals that received the inoculum of 10^6^ parasites/mL survived until the end of the experiment, including those that received the overexpressed strains, and for this reason the histological and electrocardiographic analyzes were performed only with inoculum of 10^6^ parasites/animal (par/animal) at the endpoint (30 dpi) (Figure 1a). The animals were divided into five groups (*n* = 6) as follows: Non-infected (NI), wild-type Dm28c (WT), Dm28c clone overexpressing the *TcNTPDase-1* gene (OE), Dm28c clone hemi-knockout to *TcNTPDase-1* gene (KO +/−), and the hemi-knockout Dm28c clone to *TcNTPDase-1* gene, subjected to the overexpression of this same gene (OE KO +/−) (Figure 1). The analysis of the parasitemia curve among the different studied groups showed distinct parasite loads when 10^6^ and 10^7^ par/animal inoculum were used, being as expected, higher at the 10^7^ mice group (Figure 1b,c). Furthermore, animals that received 10^6^ par/animal revealed differences in parasitemia according to the clones used (Figure 1b).

**Figure 2:**
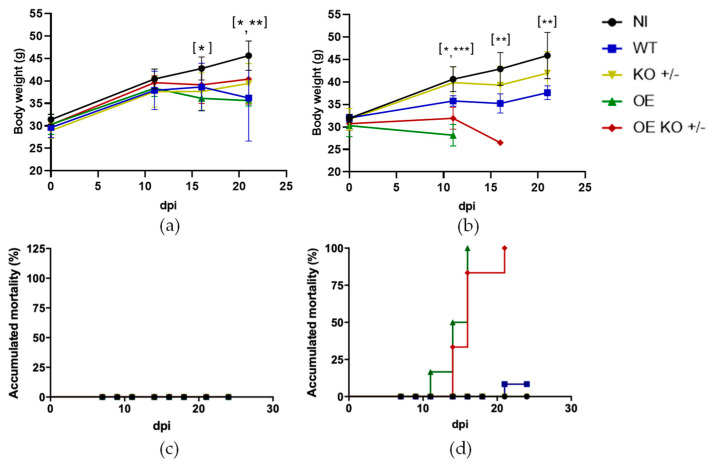
Body weight of animals infected with *T. cruzi* hemi-knockout or overexpressing the *TcNTPDase-1* gene. Mice non-infected (NI) or infected with (wild type) WT, (overexpressed) OE, (hemi-knockout) KO +/−, and (overexpressed hemi-knockout) OE KO +/− were followed up to 21 days after infection. (**a**) Body weight variation of infected mice with 10^6^ par/animal. * *p* < 0.05 at 16 dpi (NI vs. OE) and at 21 dpi (NI vs. OE KO +/−); ** *p* < 0.01 at 21 dpi (NI vs. OE). (**b**) Body weight variation of infected mice with 10^7^ par/animal. At 11 dpi * *p < 0*.05 (NI vs. WT; KO vs. WT; WT vs. OE KO; OE KO vs. OE) and *** *p* < 0.001 (NI vs. OE; NI vs. OE KO; KO vs. OE; KO vs. OE KO; WT vs. OE). At 16 dpi ** *p < 0*.01 (NI vs. WT). At 21 dpi ** *p* < 0.01 (NI vs. WT). (**c**) Monitoring of accumulated mortality in infected animals until 30 dpi with inoculum of 10^6^ par/animal, 100% survival. (**d**) Monitoring of accumulated mortality in infected animals until 30 dpi with inoculum of 10^7^ par/animal. All groups starting with *n* = 0.6. The statistical analysis was performed, as described in the Material and Methods section. At days 16 and 21, statistical analysis was not performed with OE and OE KO +/− groups, since animals died during the experiments.

The OE KO +/− showed a high peak of parasitemia, at 9 dpi. At 9 dpi, there was a significant difference between the OE KO +/− and KO +/−, which was 45.7-fold higher. In fact, the animals that received the KO +/− strain showed only a mild parasitemia peak at 7 dpi, reaching maximum of 9.67 ± 5.75 par/mL (Figure 1b). Mice inoculated with the OE strain showed an earlier peak as compared to WT (at 9 dpi) (Figure 1b). Moreover, it was possible to observe differences when we analyzed mice that received the inoculum of 10^7^ par/animal. The inoculum with WT strain peaked at 11 dpi (Figure 1c), and a higher parasite load could be noticed in the animals infected with the OE clone that peaked at 14 dpi along with the mice mortality (Figure 1c). Furthermore, animals infected with the OE strain showed higher parasitemia at 9, 11, and 14 dpi when compared with the KO +/− group (Figure 1c). Similar to what happened with the 10^6^ par/animal inoculum, the animals infected with the modified OE KO +/− strain showed two peaks of parasitemia with the first at 9 dpi (Figure 1c). The animals that received the highest inoculum of the OE and OE KO +/− strains (10^7^ par/animal) did not survive until the end of the study (Figure 2d), thus the last data for the peak of parasitemia of OE was at 14 dpi and the second peak of OE KO +/− with only one animal at 18 dpi (Figure 2b).

### 2.2. Assessment of Body Weight and Mortality

The analysis of animals that received inoculum of 10^6^ par/animals showed that animals infected with the OE strain showed weight loss, at 16 dpi, when compared to non-infected animals (Figura 2a). As the infection progressed, at 21 dpi, both overexpressed strains showed weight loss when compared to uninfected animals (Figure 2a). In the analysis of the weight data from the animals that received the inoculum of 10^7^ par/animal, we observed that at 11 dpi the animals that received the OE, OE KO +/−, and WT clones showed weight loss when compared to the NI and KO +/− animals (Figure 2b). It was observed that the animals that received the overexpressed strains (OE and OE KO +/−) showed weight loss when compared to the animals that received the WT strain. Surprisingly, the animals that received the OE strain had a significantly greater weight loss than the animals that received the OE KO +/− strain at 11 dpi. At 16 dpi, among the animals that received the inoculum of 10^7^ par/animal, we observed that the group that received the WT strain showed weight loss when compared to the NI group. However, for this inoculum (10^7^ par/animals), only groups infected with the WT and KO +/− parasites could be followed up to 21 dpi, when we observed a decrease in weight of animals that received the WT strain when compared to NI animals (Figure 2b).

All animals that received the inoculum of 10^6^ par/animal survived until the end of the experiments, regardless of the inoculated strains (Figure 2c). On the other hand, animals that received the inoculum of 10^7^ par/animal of the modified strains (OE and OE KO +/−) presented mortality of 100%, and the animals that received the WT presented mortality of 17% (Figure 2d).

### 2.3. Histopathological Analysis of Infected Animals

**Figure 3:**
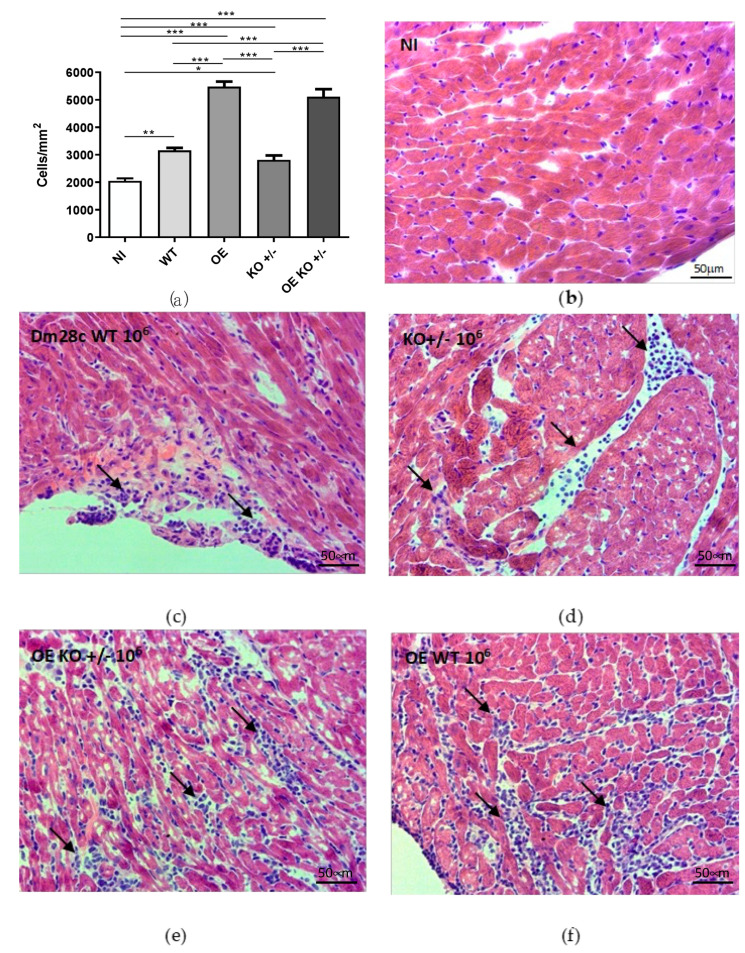
Quantitative histopathological analysis of heart sections of mice non-infected (NI) or infected with (wild type) WT, (overexpressed) OE, (hemi-knockout) KO +/−, and (overexpressed hemi-knockout) OE KO +/−. Hematoxylin and eosin stained at 30 dpi. (**a**) Quantification of the number of cell nucleus per mm^2^. (**b**–**f**) Histological sections of the animals’ hearts, demonstrating the areas of inflammatory infiltrate in the different infection groups at 30 dpi. * *p* < 0.05; ** *p* < 0.01; *** *p* < 0.001 (one-way ANOVA with Holm-Sidak post-test). Arrows indicate the carditis areas.

Since we observed 100% mortality at the OE and OE KO +/− groups in animals inoculated with 10^7^ parasites/animal at 30 dpi in the previous experiments, all the subsequent experiments were performed only with animals inoculated with 10^6^ parasites/animal. Therefore, histological sections of the heart of Swiss mice infected or not with the inoculum of 10^6^ par/animal, with wild type or recombinant parasites, were analyzed. Regarding tissue parasitism, infected cells were rare at 30 dpi in all infected groups. However, a representative image of an infected cardiac cell on the WT group is shown in Figure S1. To quantitatively analyze the inflammation in the heart tissue, the total number of cells per mm^2^ was estimated. Inflammatory infiltrates can be observed by the nuclei of cells stained in blue that appear in vessels and interstitial spaces. Previous study showed that the number of cardiac cells in a healthy mouse ventricle (129Sv/C57BL6 mice) is approximately 4000 nuclei/mm^2^ when performed in 5-µm thick slice of cardiac tissue [45]. In our study, we consider this density of nuclei comparable to the one observed in our histological sections where we quantified about 2000 nuclei/mm^2^ in thinner 3-µm slices of cardiac tissue on NI group. Increase in the number of cells per mm^2^ is linked to an increase in the heart inflammation, due to the presence of the inflammatory infiltrates. Therefore, all infected groups analyzed showed significant differences for the presence of number of cells in cardiac tissue when compared to non-infected animals (Figure 3a). Heart sections from animals that were inoculated with the WT showed a higher (1.5-fold) and significant increase in cell number, when compared to the control animals (NI) (** *p* = 0.001–Figure 3a). The animals that were infected by the OE clones (OE and OE KO +/−) showed the most significant differences between the groups analyzed, at 2.7- (*** *p* < 0.001) and 2.5-fold (*** *p* < 0.001) higher cell count, measured by the total number of cells per mm^2^, when compared to the NI group (Figure 3a). When compared to animals infected with the WT infected group, these groups (OE and OE KO +/−) showed statistically significant differences, at 1.7- (*** *p* < 0.001) and 1.6-fold higher (*** *p* < 0.001), respectively, of cells per mm^2^ (Figure 3a). Regarding animals infected with the KO +/− clones, we observed that they showed significant differences, about 1.4-fold higher (* *p* = 0.02), when compared to the control group (NI). KO +/− group presented significantly lower levels of cells per mm^2^ than OE and OE KO +/− groups (*** *p* < 0.001, for both), showing that high expression of *TcNTPDase-1* was linked to a higher inflammatory response in the heart tissues of infected animals. Representative figures of the histological sections of each group are shown in Figure 3b–f.

### 2.4. Evaluation of Electrocardiographic Changes Promoted by T. cruzi Infection

**Figure 4:**
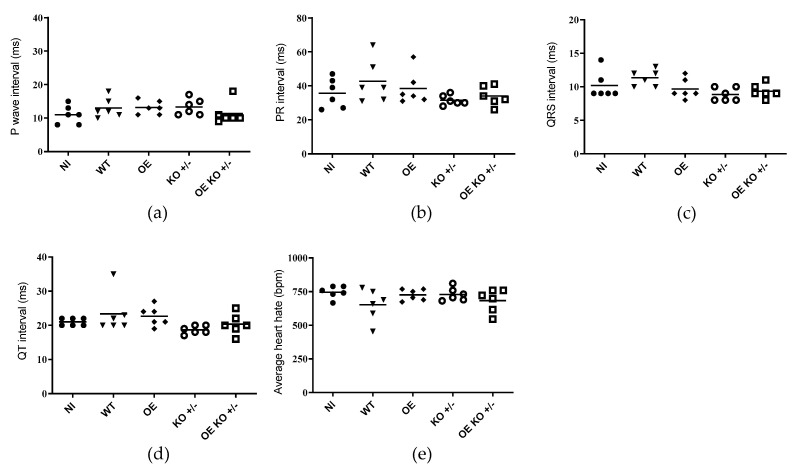
Graphical analysis of the presence of arrhythmias (individual heart rate), P wave and PR, QRS and QT intervals of mice non-infected (NI) or infected with (wild type) WT, (overexpressed) OE, (hemi-knockout) KO +/−, and (overexpressed hemi-knockout) OE KO +/− at 30 dpi. (**a**–**e**): P wave interval, PR, QRS, and QT intervals and heart rate, respectively. (ms): Milliseconds. (bpm): Beats per minute.

At present, to assess whether the changes observed have caused disturbances in the electrical conduction of the heart, an electrocardiographic study in the animals was performed (*n* = 6 per group). When analyzing the groups at 30 dpi, we were able to observe that there were no significant changes when considering the set of results between groups of animals, regarding the presence of arrhythmias (individual heart rate), P wave (the onset of atrial depolarization), QRS (the onset of ventricular depolarization), QT (time taken for ventricular depolarization and repolarization), and PR (time taken from the beginning of the P wave until the beginning of the QRS complex) intervals (Figure 4a–e).

**Figure 5:**
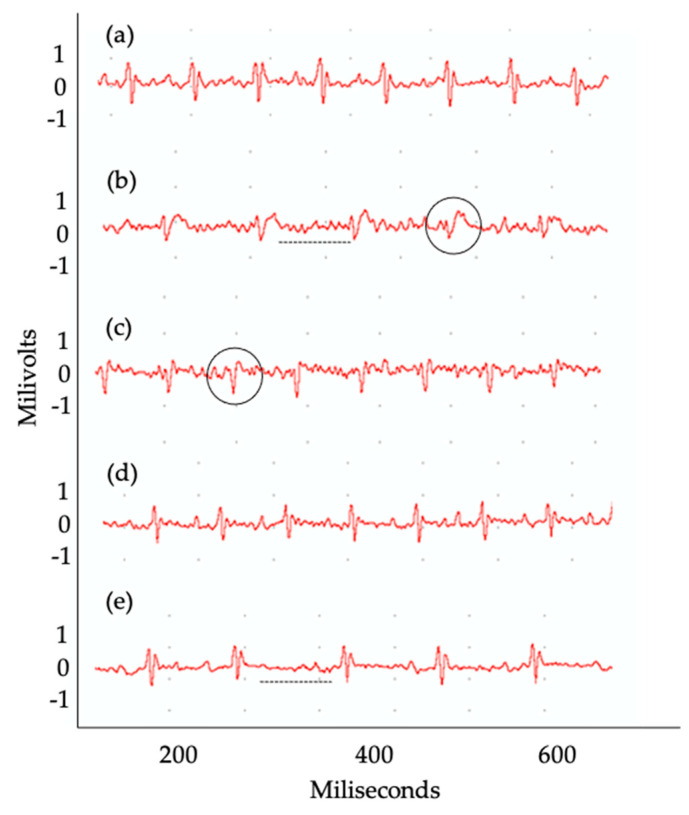
Electrocardiographic panel with examples of findings in electrocardiographic tracings in mice infected with different recombinant strains of *T. cruzi* at 30 dpi (**a**–**e**). Representative electrocardiographic tracings of animals from the following groups: (**a**) NI, (**b**) WT, (**c**) OE, (**d**) KO +/−, and OE KO +/−. Dashed lines indicate sinus bradycardia. Circles indicate atrioventricular block.

On the other hand, when groups of animals were evaluated individually, important changes in these parameters were observed. In the animals that were infected with the WT clone, 33% had sinus bradycardia and atrioventricular block. In addition, the recombinant clones overexpressing *TcNTPDase-1* gene were able to induce the presence of atrioventricular block (OE) and sinus bradycardia (OE KO +/−) in 17% of the animals, in each group (Table 1). It is important to note that although the number of animals that presented electrocardiographic alterations is not sufficient to be statistically significant within each group, the symptoms that occurred in animals presented intermediate to high parasitemia (WT, OE or OE KO +/− parasites). In Figure 5, it is possible to observe examples of the changes in the electrocardiographic tracing. Conversely, the group of animals infected with the KO +/− presented no arrhythmias or changes in the cardiac electrical conduction system at 30 dpi (Table 1 and Figure 5d). It is shown that animals infected with *T. cruzi* overexpressing the *TcNTPDase-1* gene presented electrical alterations in the heart at 30 dpi, similarly to those infected with the wild-type group. In contrast, animals infected with the parasites knocking down the *TcNTPDase-1* gene presented no arrythmias in the heart, similar to the uninfected animals.

## 3. Discussion

Even after more than 113 years of CD discovery, the mechanisms that involve the parasite’s permanence in the host organism, its infectivity and survival remain unclear [46,47]. During infectious processes, the presence of serum ATP as a result of tissue injury or lysis of infected cells, signals the formation and intensity of the immune response [48]. *T. cruzi* has a TcNTPDase on its surface that is capable of hydrolyzing ATP into ADP and the latter into AMP, preventing or hindering the formation of an effective immune response [33]. A recent study, by our group, demonstrated that the culture of VERO cells infected with a hemi-knockout clone of *T. cruzi* for the *TcNTPDase-1* gene (KO +/−) was able to decrease not only adhesion, but also the infection of these cells in this in vitro model [38]. Presently, we demonstrated that the infection of mice with *T. cruzi* hemi-knockout for *TcNTPDase-1* (KO +/−) prevented mortality, reduced the parasite load, and the percentage of inflammatory infiltrate in cardiac tissue when compared to animals infected with the wild-type clone. Furthermore, animals infected with *T. cruzi TcNTPDase-1* hemi-knockout (KO +/−) did not show any electrocardiographic changes during the 30 days of experimentation. In contrast, parasites overexpressing the *TcNTPDase-1* gene from both the wild type or (KO +/−) clones presented vastly different results, with increasing mortality, parasite load, percentage of inflammatory infiltrate, and arrythmias in the heart. It suggests that these effects are linked to the *TcNTPDase-1*, since the specific negative or positive modulation of this gene, including the (KO +/−) clone, presented reverse effects regarding the *T. cruzi* infectivity and virulence.

NTPDases have been described as being associated with several cellular functions, such as differentiation, multiplication, division, and apoptosis [33]. Present in various cell types and tissues, analogs of these enzymes are also found in the membranes of protozoan microorganisms. Although its function has not been fully discovered, several studies have investigated the participation of NTPDases in the infectivity, virulence, and survival of some parasitic protozoa [27,30,36,37,49,50,51]. At present, most of these studies have only presented data from enzymatic activities (ecto-ATPase and ecto-ADPase activities) in intact parasites or in plasma membrane fractions [25,37], investigating the role of ecto-NTPDases in the parasite–host interaction, using enzyme inhibitors and/or polyclonal antibodies [36,37]. Previous studies reported the presence of Mg^2+^-dependent-ATP-diphosphohydrolases in all forms of *T. cruzi*, but the enzymatic activity and TcNTPDase-1 mRNA levels depend on the stage of development: Both are higher in the trypomastigote and amastigote forms (infectious forms) in comparison with the epimastigote forms (non-infectious form) [15,30,31]. Since trypanosomatids do not synthesize purines, it is necessary to obtain them from exogenous sources. Then, the complete hydrolysis of ATP in adenosine comes from the action of ATP-diphosphohydrolase and ecto-5′-nucleotidase, making adenosine available for absorption [25]. As a result, it is comprehensible that amastigotes, the main replicative intracellular form of the tripanosomatids, need more nutrients (such as purines and others) to perform cellular division, expressing higher levels of ATP-diphosphohydrolases. In this case, probably the main role of these enzymes is related to the nutrient acquisition and other possible functions that are still unknown. Therefore, studies aiming at a more specific contribution of the enzyme corresponding to the *TcNTPDase-1* gene in these processes still need to be carried out, aiming at evaluating its potential as a future target for chemotherapy.

In a previous study [38], we produced recombinant clones of *T. cruzi*, hemi-knockout for the *TcNTPDase-1* gene or overexpressing this gene from the wild type or the hemi-knockout parasite, that were used to specifically evaluate the in vitro parasite-host cell interaction. We could observe that the silencing of *TcNTPDase-1* gene expression clearly affects the *T. cruzi*-VERO cells interaction, significantly decreasing the adhesion and endocytic indexes, as well as the parasitic load in the infected cells. On the other hand, *TcNTPDase-1* overexpression increased the parasite infectivity by three times. In addition, the overexpression from the hemi-knockout parasites could fully recover the *T. cruzi* infectivity and parasitic load in the infected cells, suggesting that the effects observed were specific to *TcNTPDase-1*.

Chagas disease can be caused by different discrete typing units (DTUs) which can be subdivided from TcI to TcVI and TcBat [52,53]. Different DTUs can cause different inflammatory responses in their hosts, as well as being associated with different symptoms [54]. An example is the Y strain (TcII), which is associated with the indeterminate, cardiac, and digestive forms, which in a murine model causes an early and high rate of parasitemia and mortality even in the acute phase of the disease [53,55]. Da Silva et al. (2012) [55] using BALB/c mice and inoculum of 10³ par/animal with the Y strain observed that at 24 dpi there was an accumulated mortality of 80%. Another study that sought to observe differences between infections caused by DTUs (TcI and TcII), in a canine model, noted that the infection caused by TcII was also more severe in the acute phase with a more pronounced inflammatory response than the infection caused by TcI [54]. Therefore, the choice of a DTU that causes a mild to moderate infection for assays that seek to evaluate components that can increase its virulence is extremely important. Dm28c (TcI) causes a mild infection when compared to other strains and, for this reason, requires a larger inoculum to obtain a significant symptomatology in an acute phase model.

During the validation of our experimental model, three inoculums were tested: 10^5^, 10^6^, and 10^7^ par/animal. However, during the standardization of this study, the 10^5^ par/animal inoculum did not produce parasitemia in several animals at 30 dpi, thus, we decided to use only the 10^6^ and 10^7^ par/animal inoculums afterwards. After infection, we followed the variation of body weight and cumulative mortality to assess how the parasite load among the different mutants would impact the clinical progression. We observed that animals infected with the overexpressed parasites were able to promote weight loss at 16 dpi (OE) and 21 dpi (OE and OE KO +/−) with the inoculum 10^6^ par/animal during the period of analysis when compared to uninfected animals. These data demonstrate the importance of ecto-nucleotidases present on the surface of *T. cruzi* in the course of the disease. At present, studies that sought to investigate the role of ecto-nucleotidases in the infectivity and virulence of *T. cruzi,* observed that both their overexpression and their inhibition were able to increase and decrease in the activity of the parasite, respectively [37,38]. In 2009, a study that used mice infected with blood trypomastigotes previously treated with Ecto-ATPDase inhibitors observed lower parasitemia and mortality when compared to groups that received the parasite without treatment [37]. Our data demonstrated that the overexpression of *TcNTPDase-1* was able to worsen the clinical symptoms of the disease and that even partial inhibition of this gene was sufficient to improve the clinical status of these animals.

On the other hand, although we were not able to proceed with the analysis of the groups that received the inoculum of 10^7^ par/animal, due to significant mortality after the second week, at 11 dpi we noticed that the groups that received the overexpressed strains were able to promote a greater weight loss when compared to the other groups (NI, KO +/−, and WT). In fact, a previous study that sought to observe the effects of parasite load in infection models showed that a higher load can be deleterious to the animal’s life or cause a greater severity of symptoms during infection [56]. In that study, using A/J mice and the Y strain of *T. cruzi*, authors observed that even with both inoculums used (10^3^ and 10^5^ par/animal) to receive the treatment with benznidazole in the acute phase of the disease, approximately 1 year after infection, the group that received the highest inoculum presented worse symptoms when compared to the animals that received the lowest dose. In our results, the groups that received the highest dose of inoculum, showed high rates of mortality. The accumulated mortality among animals that received 10^7^ par/animal (OE and OE KO +/− clones) was high, reaching 100% after 21 dpi. Herein, not being able to continue the analysis with these groups validates the importance of parasite load when investigating *T. cruzi* infection.

Although acute CD does not have specific symptoms, it can be present with myocarditis and electrocardiographic changes that can be fatal [42]. In experimental models, arrhythmias, atrioventricular blocks, as well as inflammatory infiltrates and the presence of amastigotes in cardiac tissue have already been described [1,42]. Based on these literature findings, our next step was to investigate the cardiac tissue of the different groups in our experimental model. For this purpose, the hearts of the animals were processed, stained, and subjected to quantification of carditis regions.

When the heart tissue sections were analyzed, we could observe the presence of inflammatory infiltrate in all infected groups. However, the frequency of inflammatory cells, present in sections of heart tissue from animals that received clones that overexpress *TcNTPDase-1* (OE and OE KO +/−) was significantly higher when compared to WT and KO +/− strains. Under normal conditions, during infectious processes, the presence of parasites in tissues is capable of promoting the attraction of inflammatory cells to the site of infection, through inflammatory mediators [57]. During the acute phase of CD, increased blood parasitemia and cardiac parasitism are common [41]. It is possible that the group of animals infected with OE and OE KO +/− clones may also have promoted an increase in the attraction of inflammatory cells to the site of infection. Surprisingly, the hemi-knockout parasites (KO +/−) showed a frequency of inflammatory infiltrate similar to the group that received the WT clone. These data might indicate that even after the silencing of one of the alleles of the *TcNTPDase-1* gene, these parasites manage to maintain their capacity for infection and virulence in their host cells, but in a reduced way, which suggests that both alleles of this gene perform the same contribution in this process.

The electrocardiographic changes promoted by the parasites hemi-knockout and overexpressing the *TcNTPDase-1* gene were also investigated, as one of the parameters was used to assess the severity of CD. Therefore, to observe whether there would be possible changes in the heart rate, P wave or in the patterns of the PR, QRS, and QT intervals of the groups infected with the different clones (WT, OE, and KO +/−) we performed an examination of ECG in these animals after 30 dpi. Regarding the analysis of the P wave, arrhythmias, and the PR, QRS, and QT intervals in the different experimental groups, we did not observe significant differences between the groups analyzed or when compared to non-infected animals during the 30 days of experimentation. However, in some individuals, we noted the presence of signs of impairment of the electrical conduction system of the heart, probably due to myocarditis. We observed that some animals showed the first signs of electrocardiographic changes, such as atrioventricular block and bradycardia in the groups infected with WT, OE, and OE KO +/− clones. The literature has shown that different models of infection can yield different results [58]. Electrocardiographic alterations will not always be present during the acute phase of the disease and studies that present some alterations during this period of infection have used strains that are naturally more virulent or even resistant to trypanocidal treatment [41,42]. Silvério et al. (2012) [41], using a chronic model of CD with *T. cruzi* Colombian strain infecting C57BL/6 mice, observed arrhythmias in infected mice at 30 dpi. However, the presence of atrioventricular block was observed only from 60 dpi. These data may suggest that more severe changes would be observed in our model if this study was carried out for a longer period. Other works have also reported the importance of the parasite’s genetic variability in producing different results for the same parameter. Santi-Roca (2017) [59] observed that BALB/c mice infected with different strains of *T. cruzi* (DTUs 1 to 6) showed variation in tissue damage and immune response between the acute and chronic phases of the infection. Another limitation of our study may have been the choice of outbred animals that, due to the fact that they are not isogenic animals and not free of specific pathogens (SPF), the genetic variability between individuals is large, allowing for greater disease resistance and requiring a greater number of animals for a robust result [60,61]. Therefore, it is possible that in our model, and with the use of outbred mice, the electrocardiographic changes observed were less pronounced and the use of a chronic phase model and isogenic mice, which has greater genetic homogeneity [62] and possibly increased susceptibility, needs to be evaluated.

Although electrocardiographic changes did not occur among our experimental groups, important changes appeared in some individuals from the groups infected with the WT, OE, and OE KO +/− clones, but not in the KO +/− group. It is possible that the increased presence of inflammatory cells in the cardiac tissue, observed in the overexpressed groups (OE and OE KO +/−) was able to induce the appearance of electrocardiographic changes at a very early stage of the disease in some individuals in these groups. On the other hand, the presence of inflammatory infiltrates was lower in animals infected with the KO +/− clone when compared to the OE and OE KO +/−, but not when compared to the WT group. This may indicate that the deletion of an allele of the *TcNTPDase-1* gene was able to promote protection in cardiac tissue and, consequently, prevent the appearance of electrical abnormalities at a very early stage of the disease.

**Figure 6:**
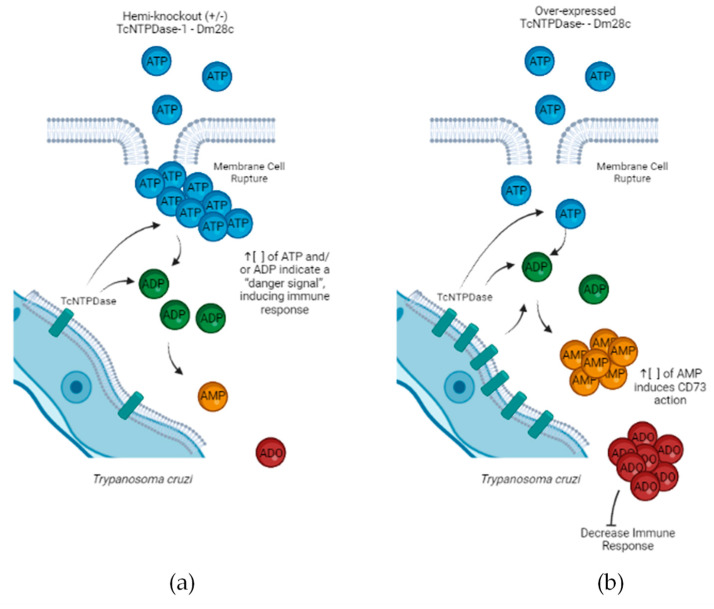
Schematic drawing of the possible action of TcNTPDase-1 during infection by recombinant *Trypanosoma cruzi*. In the scheme, the extracellular trypomastigote form is partially represented. (**a**) *T. cruzi* hemi-knockout (KO +/−) showing lower expression of TcNTPDase-1, converts a lower number of ATP molecules that signals for immune system activation. (**b**) *T. cruzi* overexpressing TcNTPDase-1 has great ability to convert ATP/ADP to AMP. Increased concentration of extracellular AMP induces ecto-5′-nucleotidase (CD73) to convert AMP to adenosine (ADO), which binds to P1 receptors decreasing the immune response.

In this scenario, contrary to what was seen with the KO +/− group, probably the overexpressing clones (OE and OE KO +/−) could be using their own ecto-nucleotidases which are capable of converting ATP/ADP to AMP, and in conjunction with the immune response, induce increased levels of adenosine in the extracellular environment, reducing the ability of the infected organism to promote an effective immune response and a parasitological elimination even in the acute phase of the disease (Figure 6).

Among the main limitations of this study, we include that all analysis were performed at the acute phase of the infection and only using male mice. Therefore, the investigation of the TcNTPDase-1 role in the establishment of the chronic Chagas cardiomyopathy (CCC), as well as in the stimulation/regulation of the immune response, is still missing. To perform this, a proper model of experimental CCC should be used, as in the infection promoted by the *T. cruzi* Colombian strain in the C57BL/6 female mice for 120 days [41]. In addition, using this model, complementary tests, such as the echocardiogram exam for an evaluation of cardiac cavities and left ventricular ejection fraction (LVEF), CK-MB levels for the characterization of cardiac damage and fibronectin assessment to evaluate fibrosis in the heart, should be performed. Although some differences in electrocardiographic alterations frequency could be observed between the parasites hemi-knockout and overexpressing *TcNTPDase-1*, these differences probably would be more conclusive if this chronic model of CCC were used. Nevertheless, our results highlight the importance of TcNTPDase-1 role in *T. cruzi* infectivity and virulence. Here, we show that the group of animals that were infected with the KO +/− parasites, which have a partial deletion of the *TcNTPDase-1* gene, had the effects of *T. cruzi* infection minimized on the severity of acute myocarditis and possibly on electrical conduction and heart rate. Due to its importance for the parasite, further studies need to be conducted, to consider the possibility of evaluating this enzyme as a new target candidate for CD chemotherapy.

## 4. Materials and Methods

### 4.1. Knockout and Overexpression of the TcNTPDase-1 Gene

The *T. cruzi* (Dm28c clone) hemi-knockout and overexpressing *TcNTPDase-1* gene from the wild type or hemi-knockout clones were produced as described in Silva-Gomes et al. (2020) [38]. Briefly, to produce the hemi-knockouts, 761 and 641 bp fragments from CDS and downstream intergenic region of the *TcNTPDase-1* gene were sequentially inserted into the pBluescript SKII (+) plasmid (Stratagene, La Jolla, CA, USA), already containing a G418 (Sigma, St. Louis, MO, USA) resistance gene cassette (pTc2KO-NTPDASE-neo). In parallel, 448 and 641 bp fragments from the upstream and downstream intergenic regions of the *TcNTPDase-1* gene were inserted in a similar plasmid but containing a hygromycin resistance gene cassette (pTc2KO-NTPDASE-hyg). The recombinant plasmids were purified by the alkaline lysis method using the plasmid mini-preparation kit (Qiagen, Hilden, Germany). The minipreparations were used for amplification of the NEO and HYG cassettes by PCR. The amplified material was purified by phenol/chloroform extraction, followed by absolute ethanol precipitation [63]. A total of 25 µg of NEO and HYG cassettes DNA, separately, were used to transfect *T. cruzi*, based on the method described by [64]. After electroporation, 500 µg/mL of G418 (Sigma, St. Louis, MO, USA) or hygromycin B (Sigma, St. Louis, MO, USA) were added to select the transfected parasites. Transfectants were cloned by serial dilution in 24-well plates. Isolates were analyzed for the correct insertion of the NEO and HYG genes into the locus of *TcNTPDase-1*.

To generate the recombinant *T. cruzi* overexpressing *TcNTPDase-1* gene, an integrative vector (pBEX v.2.0) was used, modified from the initial version [65]. The *TcNTPDase-1* gene was amplified and cloned into the vector pBEX v.2.0. Identification of the positive clones and preparation of the plasmids for transfection of *T. cruzi* were performed as described above. After electroporation, epimastigotes were cultivated in 10 mL of LIT medium (supplemented with 10,000 U of penicillin and 10 µg/mL of streptomycin–Gibco by Life Technologies, Grand Island, NY, USA). The cultures were incubated at 28 °C. After 24 h of incubation, antibiotic G418 (Sigma) at the concentration of 500 µg/mL was added. Cultures were maintained by successive passages (1:10 dilution) in LIT media supplemented with G418 (Sigma, St. Louis, MO, USA) every 8–10 days, until the absence of cell proliferation in the control culture.

### 4.2. In Vitro Metacyclogenesis and VERO Cells Infection

*Trypanosoma cruzi* epimastigotes (Dm28c clone) were cultured in LIT medium supplemented with 10% heat-inactivated bovine fetal serum at 28 °C for 5 days, to reach log-growth phase. To obtain metacyclic trypomastigotes, *T. cruzi* was allowed to be differentiated under chemically defined conditions (TAU3AAG medium), as previously described [63,66]. Briefly, epimastigotes in the late exponential growth phase were harvested from LIT medium by centrifugation and subjected to nutritional stress for 2 h in triatomine artificial urine (TAU, 190 mM NaCl, 17 mM KCl, 2 mM MgCl_2_, 2 mM CaCl_2_, 8 mM sodium phosphate buffer, pH 6.0) at a density of 5 × 10^8^ cells/mL. Then, they were transferred to cell culture flasks containing TAU3AAG (TAU supplemented with 50 mM sodium glutamate, 10 mM L-proline, 2 mM sodium aspartate, 10 mM glucose, at a density of 5 × 10^6^ cells/mL at 28 °C). After 72 h of incubation, metacyclic trypomastigotes were obtained.

To obtain cell-derived trypomastigotes, metacyclic trypomastigotes were collected as described above and used to infect VERO cells, that were grown in RPMI medium supplemented with 5% heat-inactivated bovine fetal serum (Invitrogen, Carlsbad, CA, USA), 100 U/mL penicillin, 10 µg/mL streptomycin, and 2 mM l-glutamine at 37 °C in an atmosphere of 5% CO_2_. Then, the cell monolayer was infected with metacyclic trypomastigotes (10 parasites for 1 host cell). After 24 h, the medium was discarded to remove non-internalized parasites. Thereafter, cells were washed one time with PBS and the new medium was added to the culture flasks. Cell-derived trypomastigotes were released into the supernatant 3 days after infection and were harvested by centrifugation at 3500 rpm for 10 min.

### 4.3. In Vivo Infection

#### 4.3.1. Mouse Models of Acute Infection by *T. cruzi*

For the mouse model of acute *T. cruzi* infection, male Swiss Webster mice (18 to 20 g) obtained from the animal facility’s ICTB were housed at a maximum of six per cage, maintained in a specific-pathogen-free room at 20 to 24 °C under a 12-h-light/12-h-dark cycle, and provided with sterilized water and chow ad libitum. The animals were allowed to acclimate for 7 days before the start of the experiments. Mice were infected by intraperitoneal (i.p.) injection of 10^6^ or 10^7^ cell-derived trypomastigotes of the WT, OE, KO +/− or OE KO +/− of *T. cruzi*. Age-matched non-infected mice were maintained under identical conditions (HU, 2017). At 30 dpi, the animals were submitted to electrocardiogram (ECG) before the euthanasia.

#### 4.3.2. Parasitemia and Mortality Rates

Parasitemia levels in *T. cruzi* assays were individually checked every 2 days by direct microscopic counting of parasites in 5 µL of blood obtained from the tail vein, and mortality rates were checked daily until 30 dpi and expressed as a percentage of cumulative mortality (CM) as described (Rocha-Hasler et al., 2021) [67]. To accomplish this, a Zeiss microscope model AXIO (Zeiss, Oberkochen, Germany) was used with a 40× magnification without staining the parasites.

#### 4.3.3. Electrocardiogram (ECG) Registers

All mice were intraperitoneally tranquilized with diazepam (10 mg/Kg) and the transducer was carefully placed subcutaneously according to the chosen preferential derivation (DII). Two-minute-long traces were recorded using the digital system Power Lab 2/20, which was connected to a bio-amplifier at 2 mV for 1 s (PanLab Instruments, Barcelona, Spain). Filters were standardized to between 0.1–100 Hz and traces were analyzed using the Scope software for Windows v.3.6.10 (PanLab Instruments, Spain). The ECG parameters were analyzed as previously described [41].

#### 4.3.4. Histopathology and H&E

Groups of six infected and NI control mice were euthanized under anesthesia at 30 dpi. The hearts of the mice were collected, embedded in OCT compound, cryopreserved at −80 °C, and serial cryostat sections of 3 µm-thick were fixed in cold acetone and subjected to haematoxylin and eosin staining for the evaluation of myocarditis, as previously described [68]. Images were digitized using a color view XS digital video camera adapted to a Zeiss microscope. The images were analyzed with ImageJ software (V-1.8.0_112) to quantify the cell number per mm^2^.

### 4.4. Ethics

The use of outbred stock Swiss Webster male mice and the experimental procedures were in accordance with the Brazilian Law 11.794/2008 and the regulations of the National Council for the Control of Animal Experimentation (CONCEA). All animal experimental procedures were performed following the license L-038/2017 approved by the Ethics Committee for Animal Use of Instsituto Oswaldo Cruz (CEUA/IOC). Additionally, the use of genetically-modified *T. cruzi* was approved by the Nation Council for Biosafety (CTNBio) under the license 6.146/2018 (Process 01250.040532/2018-38).

### 4.5. Statistical Analysis

The experiments were performed using six mice per group, from two independent rounds of infection that presented similar results. Data are expressed as arithmetic mean ± standard deviation. Normality distribution analysis was performed by the Shapiro-Wilk test followed by one-way ANOVA and all pairwise multiple comparisons (Tukey, Holm-Sidak or Dunn’s test) with SigmaPlot for Windows version 12.0 (Systat Software Inc., Chicago, IL, USA) were used to analyze the statistical significance of the apparent differences. Differences were considered statistically significant when *p* < 0.05, at least.

## Figures and Tables

**Table 1:** Electrocardiographic alterations frequency in animals non-infected (NI) or infected with (wild type) WT, (overexpressed) OE, (hemi-knockout) KO +/−, and (overexpressed hemi-knockout) OE KO +/−.

Groups	Arrythmias	Animals Affected (%)
Non-infected (NI)	Absent	0
WT	AVB/SB	33
OE	AVB	17
KO +/−	Absent	0
OE KO +/−	SB	17

AVB: Atrioventricular block, SB: Sinus bradycardia.

## Data Availability

All the relevant data presented in this study are available in the manuscript. The raw data are available on request from the corresponding author.

## Supplementary Materials

The following supporting information can be downloaded at: https://www.mdpi.com/article/10.3390/ijms232314661/s1.
