# Supplementary material for: Overexpression of TcNTPDase-1 Gene Increases Infectivity in Mice Infected with Trypanosoma cruzi
Source: Int J Mol Sci. 2022 Nov 24;23(23):14661. doi: 10.3390/ijms232314661 (PMC9736689; doi:10.3390/ijms232314661)
Supplement: Supplementary file 1 [file ijms-23-14661-s001.zip › ijms-1958102-supplementary.pdf]

Supplemental Figures:

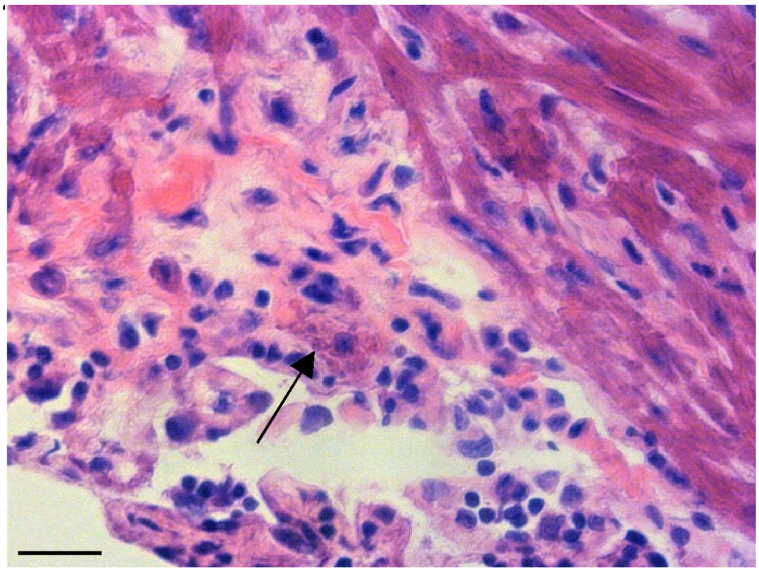

Figure S1. *Trypanosoma cruzi* infected cell in the cardiac tissue on WT group. Hematoxylin and eosin stained at 30° dpi. Arrow indicates the infected cell.
